# Retrospective observational study on the use of acetyl-l-carnitine in ALS

**DOI:** 10.1007/s00415-023-11844-6

**Published:** 2023-06-28

**Authors:** Serena Sassi, Elisa Bianchi, Luca Diamanti, Danilo Tornabene, Elisabetta Sette, Doriana Medici, Sabrina Matà, Deborah Leccese, Martina Sperti, Ilaria Martinelli, Andrea Ghezzi, Jessica Mandrioli, Valentina Virginia Iuzzolino, Raffaele Dubbioso, Francesca Trojsi, Carla Passaniti, Giulia D’Alvano, Massimiliano Filosto, Alessandro Padovani, Letizia Mazzini, Fabiola De Marchi, Lucia Zinno, Andi Nuredini, Paolo Bongioanni, Cristina Dolciotti, Elena Canali, Giulia Toschi, Antonio Petrucci, Alessia Perna, Vittorio Riso, Maurizio Inghilleri, Laura Libonati, Chiara Cambieri, Elisabetta Pupillo

**Affiliations:** 1 Istituto di Ricerche Farmacologiche Mario Negri IRCCS, Milan, Italy; 2 Neuro-Oncology Unit, IRCCS Mondino Foundation, Pavia, Italy; 3 Department of Brain and Behavioral Sciences, University of Pavia, Pavia, Italy; 4 UO di Neurologia Dipartimento Neuroscienze e Riabilitazione, Azienda Ospedaliera Universitaria di Ferrara, Ferrara, Italy; 5 Presidio Ospedaliero Fidenza AUSL Parma, Fidenza, Italy; 6 Dipartimento Neuromuscoloscheletrico e Degli Organi di Senso, Azienda Ospedaliero-Universitaria di Careggi, Florence, Italy; 7 Department of Neurosciences, Azienda Ospedaliero Universitaria di Modena, Modena, Italy; 8 Clinical and Experimental Medicine PhD Program, University of Modena and Reggio Emilia, Modena, Italy; 9 Department of Biomedical, Metabolic and Neural Sciences, University of Modena and Reggio Emilia, Modena, Italy; 10 Department of Biomedical, Metabolic and Neural Sciences, Centre for Neuroscience and Nanotechnology, University of Modena and Reggio Emilia, Modena, Italy; 11 Department of Neurosciences, Reproductive Sciences and Odontostomatology, University of Naples Federico II, Naples, Italy; 12 Dipartimento di Scienze Mediche e Chirurgiche Avanzate, Università degli Studi della Campania “Luigi Vanvitelli”, P.Zza Miraglia 2, Naples, Italy; 13 Department of Clinical and Experimental Sciences, NeMO-Brescia Clinical Center for Neuromuscular Diseases, University of Brescia, Brescia, Italy; 14 Department of Clinical and Experimental Sciences, Unit of Neurology ASST Spedali Civili Di Brescia, University of Brescia, Brescia, Italy; 15 ALS Center Azienda Ospedaliero Universitaria “Maggiore della Carità” e Università del Piemonte Orientale, Novara, Italy; 16 Department of Medicine and Surgery, University of Parma, Parma, Italy; 17 Dpt. Medical Specialties, Azienda Ospedaliero-Universitaria Pisana, Pisa, Italy; 18 U.O di Neurologia, Presidio Ospedaliero S.Maria Nuova Azienda USL, IRCCS di Reggio Emilia, Florence, Italy; 19 Center for Neuromuscular and Neurological Rare Diseases, San Camillo Forlanini Hospital, Rome, Italy; 20 Dipartimento di Neuroscienze Umane, Università di Roma “Sapienza” UOSD Malattie Neurodegenerative-Centro Malattie Rare Neuromuscolari, Policlinico Universitario Umberto I, Rome, Italy

**Keywords:** Observational study, Treatment, Amyotrophic lateral sclerosis, Neurological disease, Case–control, Efficacy

## Abstract

ALCAR (Acetyl-L-carnitine) is a donor of acetyl groups and increases the intracellular levels of carnitine, the primary transporter of fatty acids across the mitochondrial membranes. In vivo studies showed that ALCAR decrease oxidative stress markers and pro-inflammatory cytokines. In a previous double-blind placebo-controlled phase II trial showed positive effects on self-sufficiency (defined as a score of 3+ on the ALSFRS-R items for swallowing, cutting food and handling utensils, and walking) ALSFRS-R total score and FVC. We conducted an observational, retrospective, multicentre, case–control study to provide additional data on the effects of ALCAR in subjects with ALS in Italy. Subjects treated with ALCAR 1.5 g/day or 3 g/day were included and matched with not treated subjects by sex, age at diagnosis, site of onset, and time from diagnosis to baseline, (45 subjects per group). ALCAR 3 g/day vs not treated: 22 not treated subjects (48.9%) were still alive at 24 months after baseline, compared to 23 (51.1%) treated subjects (adj. OR 1.18, 95% CI 0.46–3.02). No statistically significant differences were detected in ALSFRS nor FVC nor self-sufficiency. ALCAR 1.5 g/day vs not treated: 22 not treated subjects (48.9%) were still alive at 24 months after baseline, compared to 32 (71.1%) treated subjects (adj. OR 0.27, 95% CI 0.10–0.71). For ALSFRS-R, a mean slope of − 1.0 was observed in treated subjects compared to − 1.4 in those not treated (*p* = 0.0575). No statistically significant difference was detected in the FVC nor self-sufficiency. Additional evidence should be provided to confirm the efficacy of the drug and provide a rationale for the dosage.

## Introduction

Amyotrophic lateral sclerosis (ALS) is a neurodegenerative disease resulting in progressive weakness and selective degeneration of the motor neurons, leading to death from respiratory failure within 3–4 years [1].

Acetyl-l-carnitine (ALCAR) counteracts motor-neuron death induced by toxic agents or deprivation of trophic factors and slows disease progression in animal models [2–4]. The drug also ameliorates mitochondrial dysfunction [5], restores synaptic transmission [6] and exerts protective effects against neuroinflammation [7]. As a consequence, decreased levels of oxidative stress markers and pro-inflammatory cytokines have been detected [8, 9]. In a previous double-blind placebo-controlled phase II trial, 42 self-sufficient subjects (with a score of 3+ on the ALSFRS-R items for swallowing, cutting food and handling utensils, and walking) with ALS [10] received ALCAR 3 g/day and 40 received placebo in an equivalent dose, and were followed for 12 months or until death [11]. In the Intention to treat (ITT) population, 80.9% of subjects receiving ALCAR and 97.5% of those receiving placebo became non-self-sufficient (*p* = 0.0296). Based on previous data coming from preclinical and clinical studies and its excellent clinical safety profile, ALCAR is a promising treatment for ALS. However, the efficacy of the drug has yet to be confirmed by further studies.

For this reason, we conducted an observational, retrospective, multicentre, case–control study to add more evidence to the results of the pilot study.

## Methods

### Settings and study population

Subjects were identified retrospectively from medical records of 14 participating Italian sites (representative of the entire peninsula) according to the following inclusion criteria: aged 18+ years at ALS diagnosis, definite or probable or probable laboratory-supported ALS according to the revised El Escorial diagnostic criteria [12]; self-sufficiency; satisfactory respiratory function (FVC ≥ 80% of predicted).

Possible and suspected ALS were not included to avoid misclassification bias.

The main exclusion criteria were: antecedent polio infection; other motor neuron disease; involvement of other systems possibly determining a functional impairment; other severe clinical conditions; participation in a clinical trial.

Given that ALS is an incurable disease, the health care system of several Italian regions may support, after approval from the regional rare diseases technical group, off-label use of selected treatments with a promising evidence reported in preliminary early clinical studies. In this setting, since 2011 ALCAR has been prescribed by some specialized ALS centres in Italy, without any additional cost for people living with ALS.

Subjects were selected from participant sites that prescribe ALCAR 3 or 1.5 g/day per OS as per clinical practice. Only subjects that started for the first time to use ALCAR (for a minimum period of 6 months) from January 1st, 2011 to April 30th, 2019 were included. Controls were subjects not treated with ALCAR, selected with the same inclusion and exclusion criteria as treated subjects.

Subjects treated with ALCAR and controls were matched 1:1 by sex, age at diagnosis (± 5 years), site of onset (spinal/bulbar), and time from diagnosis to baseline (± 3 months). Baseline was defined as the start date of ALCAR treatment for treated subjects. For controls, baseline was defined as the date of the visit in which the disease duration was nearest to the disease duration of the matched treated subject. All available subjects in participating centres were initially included in the study and matching was subsequently performed retaining in the analyses only matched subjects.

Each subject was retrospectively followed for a minimum of 24 months or until death and for a maximum period of 10 years.

### Sample size calculation

In a phase II trial [11], the estimated percentage of subjects alive after 24 months was 64% among those receiving ALCAR (3 g/die) and 34% among those receiving placebo (absolute difference: 30%). A total of 45 subjects treated with ALCAR 3 g/day and 45 subjects in the control group is needed to detect this difference with 80% power and 5% level of significance. The minimum sample size to be reached after matching is of 45 subjects treated with ALCAR 3 g/day and 45 matched controls (1:1 matching).

To have the same power to detect the same effect for subject treated with ALCAR 1.5 g/day 45 additional pairs are needed.

### Primary endpoint and related outcome measure

The proportion of subjects alive at 24 months after baseline among subjects treated with ALCAR 3 g/day compared to not treated subjects.

### Secondary endpoints and related outcome measures

1. The cumulative probability of survival during the available follow-up period, in subjects treated with ALCAR 3 g/day and not treated subjects.
2. The mean change of ALSFRS-R total score during the 12 months after baseline, in subjects treated with ALCAR 3 g/day and not treated subjects.
3. The mean change of FVC score during the 12 months after baseline, in subjects treated with ALCAR 3 g/day and not treated subjects.
4. The proportion of subjects becoming non-self-sufficient after 12 months of follow-up, defined as those scoring 2 or lower on at least one of the ALSFRS-R items for swallowing, and/or cutting food and/or handling utensils, or walking, in subjects treated with ALCAR 3 g/day and not treated subjects.
5. The cumulative probability of losing self-sufficiency during the 12 months after baseline, considering subjects with ALSFRS-R data recorded at least every 3 months, in subjects treated with ALCAR 3 g/day and not treated subjects.
6. The mean change of ALSFRS-R total score during the 18 and 24 months after baseline, in subjects treated with ALCAR 3 g/day and not treated subjects.
7. The mean change of FVC score during the 18 and 24 months after baseline, in subjects treated with ALCAR 3 g/day and not treated subjects.

### Secondary exploratory endpoints and related outcome measures

The proportion of subjects alive at 24 months after baseline in subjects treated with ALCAR 1.5 g/day and not treated subjects. The same as in secondary endpoints and related outcomes 1–7, but considering subjects treated with ALCAR 1.5 g/day compared to not treated subjects.

### Data collection

We collected demographic and clinical data at baseline (El Escorial category, date of disease onset and diagnosis, site of onset), medical/surgical history, including comorbidities, physical and neurological examination.

To assess changes in functional impairment and vital capacity, ALSFRS-R scale and FVC were recorded at every available visit for 12 months, collecting all available data reported in the medical records (maximum once per month, 12 times per subject). If available, data were also recorded at 18 and 24 months after baseline. Any change in concomitant therapy, medical history or disease progression was also collected at each available follow-up. For date of death information, we asked investigators to collect death certificate.

Given the retrospective data collection in subjects diagnosed between 2011 and 2019, neither genetic data nor biological samples were.

An electronic, centralized, validated and password protected CRF according to the European privacy law (General Data Protection Regulation UE n. 2016/679), was used. Anonymized data were entered into the eCRFs by site staff.

This study was planned and performed according to the principles of Good Clinical Practice (ICH-GCP), the declaration of Helsinki, and the national laws and regulations about clinical studies. The study received approval from the Independent Ethics Committee (IEC) of the Promoter and of each involved site.

### Statistical analysis

Descriptive statistics on demographic and clinical variables were reported in subjects treated with ALCAR 3 g/day and not treated matched controls. Treated and not treated subjects were compared using the chi-square or the Fisher’s exact test for categorical variables, and the Wilcoxon–Mann–Whitney test for continuous variables. The number and percentage of subjects alive at 24 months after baseline were calculated and compared between treatment groups using the chi-square test. Univariable and multivariable logistic regression models were used to calculate the odds ratio (OR) and adjusted OR (adj. OR) for the risk of death within 24 months after baseline. The progression rate was calculated as (48 − ALSFRS-R at baseline)/(disease duration at baseline), and subjects were categorized as fast progressors (rate > 0.67) or slow progressors (rate ≤ 0.67) [13]. The cumulative probability of survival over 24 months of follow-up in each treatment group was estimated using Kaplan–Meier survival curves and compared between treatment groups using the log-rank test. Univariable and multivariable Cox’s proportional hazards models were used to estimate the hazard ratio (HR) and adjusted HR (adj. HR) for death. The progression over 24 months of follow-up of ALSFRS-R total score and FVC% were evaluated using repeated measures linear mixed models with random intercept and slope, with, separately, ALSFRS and FVC% as dependent variable and treatment (not treated or treated with ALCAR), time (month 0–24) and treatment × time interaction as independent variables. The number and percentage of self-sufficient subjects at 12 months was calculated and compared between treatment groups using the Chi-square test. Univariable and multivariable logistic regression models were used to calculate the OR and adj. OR for the risk of losing self-sufficiency within 24 months after baseline. The cumulative probability of remaining self-sufficient was estimated using Kaplan–Meier survival curves. Univariable and multivariable Cox’s proportional hazards models were used to estimate the HR and adj. HR.

All multivariable models were adjusted for El Escorial category, use of riluzole and progression rate category.

The entire statistical analysis plan was repeated in subjects treated with ALCAR 1.5 g/day and their matched controls as a secondary exploratory analysis. Missing data were handled using the listwise deletion in logistic regression and Cox models. In linear mixed models all available time points were included for each subject. The significance level was set to 0.05. Analyses were performed using the SAS software, version 9.4 (SAS Institute, Cary, NC, USA).

A post-hoc sensitivity analysis using a propensity score matching was also performed to evaluate the impact of a different matching approach on the estimated effects of treatment with ALCAR. The propensity score for receiving ALCAR treatment was calculated in subjects treated with 3 g/day and not treated, and, separately, in those treated with 1.5 g/day and not treated. Logistic regression models were used to calculate propensity scores, including as covariates: sex, onset, El Escorial category, use of riluzole, ALSFRS-R total score at baseline, FVC at baseline, time from diagnosis to the first available visit for not treated subjects or to the date of ALCAR treatment start for treated subjects, diagnostic delay (time from onset to diagnosis), age at baseline. A 1:1 matching on propensity score (± 0.02) of subjects treated with ALCAR 3 g/day with not treated subjects, and, separately, of subjects treated with ALCAR 1.5 g/day with not treated subjects, was then performed using the same pool of not treated subjects.

## Results

**Fig. 1:**
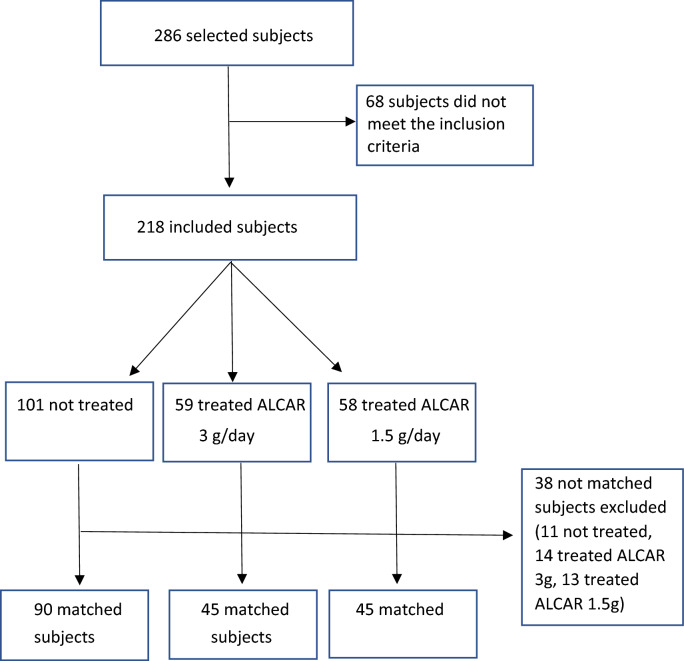
Study flow chart

We collected data of 286 subjects. 68 subjects did not meet the inclusion criteria and were excluded, leading to 218 subjects available for matching (101 not treated, 59 treated with ALCAR 3 g/day and 58 treated with ALCAR 1.5 g/day). We performed a 1:1 matching of subjects treated with ALCAR 3 g/day with 45 not treated subjects and separately a 1:1 matching of subjects treated with ALCAR 1.5 g/day with 45 additional not treated subjects. 38 subjects were not matched (11 not treated, 14 treated with ALCAR 3 g/day and 13 treated with ALCAR 1.5 g/day and were excluded from the analyses (Fig. 1).

All subjects were treated until the end of follow-up.

Descriptive statistics comparing subjects treated with ALCAR 3 g/day and not treated matched controls are shown in Table 1. Treated and not treated subjects were comparable for most baseline characteristics. Differences in baseline characteristics were observed for the El Escorial category that was more frequently probable laboratory supported in not treated (47%) than in treated (24%) subjects, while treated were more frequently definite ALS (36%) as compared to not treated (16%). All subjects treated with ALCAR were concomitantly treated with riluzole, while about 7% of those not treated with ALCAR did not take riluzole. Progression rate was fast in 24% of treated and 31% of not treated subjects. The baseline ALSFRS-R total score and FVC% were comparable in the 2 groups.

**Table 1:** Descriptive statistics in treated with ALCAR 3 g/day and matched not treated

	Not treated		Treated with ALCAR 3 g/day		*p* value
	*n*	%	*n*	%	
Sex					> 0.9999
Female	14	31.1	14	31.1	
Male	31	68.9	31	68.9	
Site of onset					> 0.9999
Bulbar	14	31.1	14	31.1	
Spinal	31	68.9	31	68.9	
El Escorial category					0.0355
Definite	7	15.6	16	35.6	
Probable	17	37.8	18	40.0	
Probable laboratory supported	21	46.7	11	24.4	
Riluzole use					0.2416
Yes	42	93.3	45	100.0	
No	3	6.7	0	0.0	
Progression rate					0.4802
Fast	14	31.1	11	24.4	
Slow	31	68.9	34	75.6	

	Not treated		Treated with ALCAR 3 g/day		*p* value
	Median	IQR	Median	IQR	
Age onset	66.1	60.5–70.8	65.2	60.1–71.1	0.9005
Age diagnosis	67.0	61.1–71.1	66.2	60.6–71.6	0.9103
Diagnostic delay	8.2	4.8–12.1	9	4.3–12.1	0.8816
Progression rate at diagnosis	0.37	0.25–0.74	0.47	0.24–0.67	0.6124
Follow up duration	28.9	20.5–42.9	25.9	19.2–42.0	0.8941
Age baseline	67.0	61.3–71.1	66.5	60.9–71.6	0.8627
Time from diagnosis to baseline (months)	0.3	0.0–1.0	0.2	0.0–1.4	0.9447
ALSFRS-R	45	43–45	44	42–45	0.3186
FVC %	96	88–103	96	88–107	0.9326

*ALCAR* acetyl-l-carnitine, *FVC* forced vital capacity, *ALSFRS-R* amyotrophic lateral sclerosis functional rating scale-revised

### Primary endpoint

**Fig. 2:**
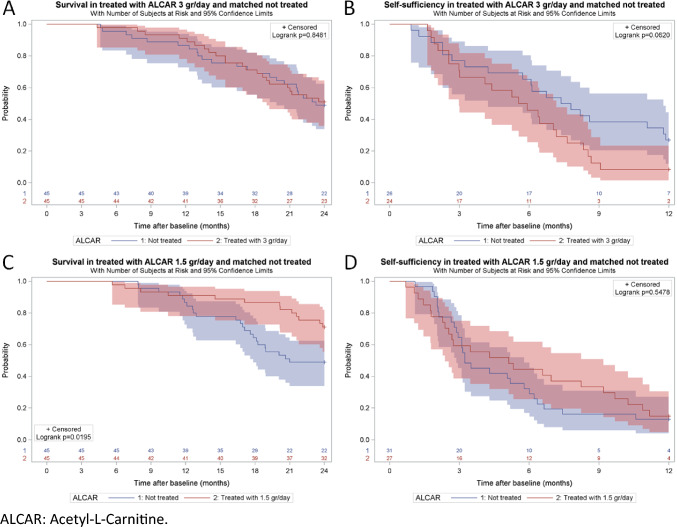
Cumulative survival probability and cumulative probability of remaining self-sufficient in treated with ALCAR and not treated

Among not treated subjects 22 (48.9%) were still alive at 24 months after baseline, compared to 23 (51.1%) among treated subjects (*p* = 0.8330, adj. OR 1.18, 95% CI 0.46–3.02). The cumulative survival probability (Fig. 2A) was 0.96 at 6 months, 0.87 at 12 months, 0.71 at 18 months, and 0.49 at 24 months in not treated subjects. The corresponding numbers in treated subjects were 0.98, 0.91, 0.71 and 0.51 (*p* = 0.8481, adj. HR 1.15, 95% CI 0.59–2.21) (Table 2).

**Table 2:** Risk of death and risk of losing self-sufficiency in treated with ALCAR 3 g/day and not treated: univariable and multivariable analyses

Status at 24 months from baseline	Not treated		Treated with ALCAR 3 g/day		*p* value	Univariable analysis^a^		Multivariable analysis^b^	
	*n*	%	*n*	%		OR	95% CI	adj. OR	95% CI
Alive	22	48.9	23	51.1	0.8330	0.91	0.40–2.09	1.18	0.46–3.02
Dead	23	51.1	22	48.9					

Survival over 24 months of follow-up	Number at risk	Survival probability	Number at risk	Survival probability	*p* value	HR	95% CI	adj. HR	95% CI
0 months	45	1.00	45	1.00	0.8481	0.95	0.53–1.70	1.15	0.59–2.21
3 months	45	1.00	45	1.00					
6 months	43	0.96	44	0.98					
9 months	40	0.89	42	0.93					
12 months	39	0.87	41	0.91					
15 months	34	0.76	36	0.80					
18 months	32	0.71	32	0.71					
21 months	28	0.62	27	0.60					
24 months	22	0.49	23	0.51					

Self-sufficiency at 12 months from baseline	*n*	%	*n*	%	*p* value	OR	95% CI	adj. OR	95% CI
Self-sufficient	7	16.7	2	4.7	0.0719	4.1	0.79–21.02	7.96	1.08–58.95
Not self-sufficient	35	83.3	41	95.3					
Unknown	3		2						

Self-sufficiency over 12 months of follow-up	Number at risk	Probability of remaining self-sufficient	Number at risk	Probability of remaining self-sufficient	*p* value	HR	95% CI	adj. HR	95% CI
0 months	26	1.00	24	1.00	0.0620	1.8	0.96–3.37	2.02	0.99–4.12
3 months	20	0.77	17	0.71					
6 months	17	0.65	11	0.46					
9 months	10	0.38	3	0.13					
12 months	7	0.27	2	0.08					

*ALCAR* acetyl-l-carnitine, *OR* odds ratio, *adj. OR* adjusted odds ratio, *HR* hazard ratio, *adj. HR* adjusted hazard ratio

^a^Matched by age (± 5 years), sex, disease duration (± 3 years), site of onset

^b^Matched by age (± 5 years), sex, disease duration (± 3 years), site of onset; adjusted by riluzole use, EL Escorial category, progression rate category

### Secondary endpoints

At 12 months after baseline 7 not treated subjects (16.7%) were self-sufficient, as compared to 2 (4.7%) treated subjects (*p* = 0.0719, adj. OR 7.96, 95% CI 1.08–58.95). The cumulative probability of remaining self-sufficient (Fig. 2B) was 0.77 at 3 months, 0.65 at 6 months, 0.38 at 9 months, and 0.27 at 12 months in not treated subjects. The corresponding numbers in treated subjects were 0.71, 0.46, 0.13 and 0.08 (*p* = 0.0620, adj. HR 2.02, 95% CI 0.99–4.12) (Table 2).

**Fig. 3:**
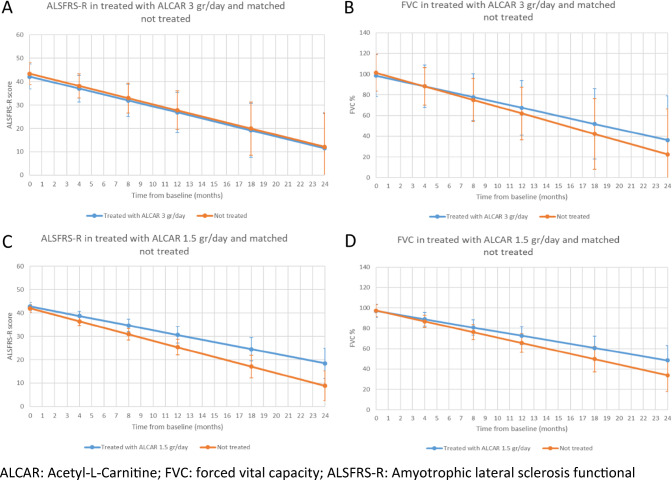
Progression of ALSFRS-R and FVC% in treated with ALCAR and not treated

In treated subjects, the mean estimated ALSFRS-R total score was 42.2 at baseline, 26.9 at month 12 and 11.5 at month 24, with a mean slope of − 1.3, as compared to 43.4, 27.7 and 12.1, with a mean slope of − 1.3 in not treated subjects (slope difference: 0.0; 95% CI − 0.4, 0.4; *p* = 0.8910) (Table 3) (Fig. 3A).

**Table 3:** Progression of ALSFRS-R and FVC% in treated with ALCAR 3 g/day and not treated

Group	Time	ALSFRS-R				FVC			
		Estimate^a^	95% lower CL	95% upper CL	*p* value	Estimate^a^	95% lower CL	95% upper CL	*p* value
Treated with ALCAR 3 g/day	Month 0	42.2	39.5	44.8		98.6	88.5	108.7	
	Month 4	37.1	34.2	39.9		88.2	78.0	98.4	
	Month 8	32.0	28.5	35.4		77.8	66.5	89.2	
	Month 12	26.9	22.6	31.2		67.4	54.2	80.7	
	Month 18	19.2	13.4	25.0		51.8	34.8	68.8	
	Month 24	11.5	4.2	18.9		36.2	14.9	57.6	
	Slope	− 1.3	− 1.6	− 1.0	< 0.0001	− 2.6	− 3.4	− 1.8	< 0.0001
Not treated	Month 0	43.4	41.0	45.8		101.4	92.5	110.2	
	Month 4	38.2	35.6	40.7		88.2	79.3	97.2	
	Month 8	33.0	29.7	36.2		75.1	64.7	85.5	
	Month 12	27.7	23.6	31.9		61.9	49.2	74.7	
	Month 18	19.9	14.3	25.6		42.2	25.2	59.3	
	Month 24	12.1	4.8	19.4		22.5	0.6	44.4	
	Slope	− 1.3	− 1.6	− 1.0	< 0.0001	− 3.3	− 4.2	− 2.4	< 0.0001
Treated with ALCAR 3 g/day vs. not treated (difference)	Month 0	− 1.2	− 2.9	0.5		− 2.8	− 9.7	4.1	
	Month 4	− 1.1	− 3.4	1.1		0.0	− 7.3	7.3	
	Month 8	− 1.0	− 4.6	2.5		2.7	− 7.6	13.1	
	Month 12	− 0.9	− 6.0	4.2		5.5	− 9.0	20.0	
	Month 18	− 0.7	− 8.2	6.8		9.6	− 11.8	31.0	
	Month 24	− 0.5	− 10.5	9.4		13.7	− 14.8	42.2	
	Slope	0.0	− 0.4	0.4	0.8910	0.7	− 0.6	1.9	0.2747

*ALCAR* acetyl-l-carnitine, *FVC* forced vital capacity, *ALSFRS-R* amyotrophic lateral sclerosis functional rating scale-revised, *CL* confidence limit

^a^Matched by age (± 5 years), sex, disease duration (± 3 years), site of onset; adjusted by riluzole use, EL Escorial category, progression rate category

The mean estimated FVC% was 98.8 at baseline, 67.4 at month 12 and 36.2 at month 24 in treated subjects, with a mean slope of − 2.6, as compared to 101.4, 61.9 and 22.5 in not treated subjects, with a mean slope of − 3.3 (slope difference: 0.7; 95% CI − 0.6, 1.9; *p* = 0.2747) (Table 3) (Fig. 3B).

### Secondary exploratory analysis

A total of 45 subjects treated with ALCAR 1.5 g/day and 45 matched controls were included in this secondary exploratory analysis. Descriptive statistics comparing subjects treated with ALCAR 1.5 g/day and not treated matched controls are shown in Table 4. Treated and not treated subjects were comparable for most baseline characteristics. Differences in baseline characteristics were observed for the definite El Escorial category that was more frequent in treated subjects (36% vs. 13%), while the probable and probable laboratory supported categories were less frequent in treated subjects (33% vs. 40% and 31% vs. 46%, respectively). All except one subject treated with ALCAR were concomitantly treated with riluzole, while 11% of those not treated with ALCAR did not take riluzole. Progression rate was fast in 15% of treated and 29% of not treated subjects. The baseline ALSFRS-R total score and FVC% were comparable in the 2 groups.

**Table 4:** Descriptive statistics in treated with ALCAR 1.5 g/day and matched not treated

	Not treated		Treated with ALCAR 1.5 g/day		*p* value
	*n*	%	*n*	%	
Sex					> 0.9999
Female	11	24.4	11	24.4	
Male	34	75.6	34	75.6	
Site of onset					> 0.9999
Bulbar	13	28.9	13	28.9	
Spinal	32	71.1	32	71.1	
El Escorial category					0.0408
Definite	6	13.3	16	35.6	
Probable	18	40.0	15	33.3	
Probable laboratory supported	21	46.7	14	31.1	
Riluzole use					0.0910
Yes	40	88.9	44	97.8	
No	5	11.1	1	2.2	
Progression rate					0.1282
Fast	13	28.9	7	15.6	
Slow	32	71.1	38	84.4	

	Not treated		Treated with ALCAR 1.5 g/day		*p* value
	Median	IQR	Median	IQR	
Age onset	64.8	55.9–71.4	63.8	56.1–69.1	0.8155
Age diagnosis	65.4	56.8–71.9	64.9	57.8–70.1	0.9295
Diagnostic delay	8.3	5.1–10.8	10.6	5.0–14.3	0.0913
Follow up duration	25.8	18.9–49.6	33.6	22.4–47.7	0.2424
Progression rate at diagnosis	0.46	0.28–0.74	0.28	0.15–0.52	0.0067
Age baseline	65.4	56.8–72.0	65.0	57.8–70.1	0.9359
Time from diagnosis to baseline (months)	0.0	0.0–0.9	0.0	0.0–0.0	0.4410
ALSFRS-R	44	41–46	45	44–46	0.0614
FVC %	96	93–105	97	89–102	0.4146

*ALCAR* acetyl-l-carnitine, *FVC* forced vital capacity, *ALSFRS-R* amyotrophic lateral sclerosis functional rating scale-revised

#### Primary endpoint in treated with ALCAR 1.5 g/day

Among not treated subjects 22 (48.9%) were still alive at 24 months after baseline, as compared to 32 (71.1%) among treated subjects (*p* = 0.0314, adj. OR 0.27, 95% CI 0.10–0.71). The cumulative survival probability (Fig. 2C) was 1.00 at 6 months, 0.87 at 12 months, 0.64 at 18 months, and 0.49 at 24 months in not treated subjects. The corresponding numbers in treated subjects were 0.98, 0.91, 0.88 and 0.71 (*p* = 0.0195, adj. HR 0.34, 95% CI 0.16–0.71) (Table 5).

**Table 5:** Risk of death and risk of losing self-sufficiency in treated with ALCAR 1.5 g/day and not treated: univariable and multivariable analyses

Status at 24 months from baseline	Not treated		Treated with ALCAR 1.5 g/day		*p* value	Univariable analysis^a^		Multivariable analysis^b^	
	*n*	%	*n*	%		OR	95% CI	adj. OR	95% CI
Alive	22	48.9	32	71.1	0.0314	0.39	0.16–0.93	0.27	0.10–0.71
Dead	23	51.1	13	28.9					

Survival over 24 months of follow-up	Number at risk	Survival probability	Number at risk	Survival probability	*p* value	HR	95% CI	adj. HR	95% CI
0 months	45	1.00	45	1.00	0.0195	0.45	0.23–0.90	0.34	0.16–0.71
3 months	45	1.00	45	1.00					
6 months	45	1.00	44	0.98					
9 months	43	0.96	42	0.93					
12 months	39	0.87	41	0.91					
15 months	35	0.78	40	0.89					
18 months	29	0.64	39	0.87					
21 months	22	0.49	37	0.82					
24 months	22	0.49	32	0.71					

Self-sufficiency at 12 months from baseline	*n*	%	*n*	%	*p* value	OR	95% CI	adj. OR	95% CI
Self-sufficient	4	9.5	3	7.7	> 0.9999	1.26	0.26–6.04	1.47	0.27–7.86
Not self-sufficient	38	90.5	36	92.3					
Unknown	3		6						

Self-sufficiency over 12 months of follow-up	Number at risk	Probability of remaining self-sufficient	Number at risk	Probability of remaining self-sufficient	*p* value	HR	95% CI	adj. HR	95% CI
0 months	31	1.00	27	1.00	0.5478	0.84	0.48–1.48	0.89	0.47–1.69
3 months	20	0.64	16	0.59					
6 months	10	0.32	12	0.44					
9 months	5	0.16	9	0.33					
12 months	4	0.13	4	0.15					

*ALCAR* acetyl-l-carnitine, *OR* odds ratio, *adj. OR* adjusted odds ratio, *HR* hazard ratio, *adj. HR* adjusted hazard ratio

^a^Matched by age (± 5 years), sex, disease duration (± 3 years), site of onset

^b^Matched by age (± 5 years), sex, disease duration (± 3 years), site of onset; adjusted by riluzole use, EL Escorial category, progression rate category

#### Secondary endpoints in treated with ALCAR 1.5 g/day

At 12 months after baseline 4 not treated subjects (9.5%) were self-sufficient, as compared to 3 (7.7%) treated subjects (*p* = 0.9999, adj. OR 1.47, 95% CI 0.27–7.86). The cumulative probability of remaining self-sufficient (Fig. 2D) was 0.64 at 3 months, 0.32 at 6 months, 0.16 at 9 months, and 0.13 at 12 months in not treated subjects. The corresponding numbers in treated subjects were 0.59, 0.44, 0.33 and 0.15 (*p* = 0.5478, adj. HR 0.84, 95% CI 0.48–1.48) (Table 5).

The mean estimated ALSFRS-R total score was 42.8 at baseline, 30.6 at month 12 and 18.4 at month 24 in treated subjects, with a mean slope of − 1.0, as compared to 41.9, 25.4 and 8.8 in not treated subjects, with a mean slope of − 1.4 (slope difference: 0.4; 95% CI 0.0, 0.7; *p* = 0.0575) (Table 6) (Fig. 3C). The mean estimated FVC% was 97.0 at baseline, 72.7 at month 12 and 48.4 at month 24 in treated subjects, with a mean slope of − 2.0, as compared to 97.4, 65.8 and 33.8 in not treated subjects, with a mean slope of − 2.6 (slope difference: 0.6; 95% CI − 0.2, 1.5; *p* = 0.1542) (Table 6) (Fig. 3D).

**Table 6:** Progression of ALSFRS-R and FVC% in treated with ALCAR 1.5 g/day and not treated

Group	Time	ALSFRS-R				FVC			
		Estimate^a^	95% lower CL	95% upper CL	*p* value	Estimate^a^	95% lower CL	95% upper CL	*p* value
Treated with ALCAR 1.5 g/day	Month 0	42.8	41.1	44.5		97.0	90.6	103.3	
	Month 4	38.7	36.8	40.7		88.9	82.3	95.4	
	Month 8	34.7	32.0	37.3		80.8	73.3	88.2	
	Month 12	30.6	27.1	34.1		72.7	63.8	81.5	
	Month 18	24.5	19.5	29.5		60.6	49.1	72.0	
	Month 24	18.4	11.9	25.0		48.4	34.0	62.9	
	Slope	− 1.0	− 1.3	− 0.7	< 0.0001	− 2.0	− 2.6	− 1.5	< 0.0001
Not treated	Month 0	41.9	40.4	43.5		97.4	91.6	103.1	
	Month 4	36.4	34.6	38.2		86.8	80.7	92.8	
	Month 8	30.9	28.4	33.4		76.2	68.9	83.5	
	Month 12	25.4	22.0	28.8		65.6	56.5	74.7	
	Month 18	17.1	12.2	21.9		49.7	37.3	62.1	
	Month 24	8.8	2.5	15.2		33.8	17.9	49.8	
	Slope	− 1.4	− 1.6	− 1.1	< 0.0001	− 2.6	− 3.3	− 2.0	< 0.0001
Treated with ALCAR 1.5 g/day vs. not treated (difference)	Month 0	0.9	− 0.6	2.3		− 0.4	− 5.8	5.0	
	Month 4	2.3	0.4	4.3		2.1	− 3.8	8.0	
	Month 8	3.8	0.6	6.9		4.6	− 3.4	12.6	
	Month 12	5.2	0.7	9.8		7.1	− 3.7	17.9	
	Month 18	7.4	0.7	14.2		10.8	− 4.7	26.3	
	Month 24	9.6	0.7	18.6		14.6	− 5.8	35.0	
	Slope	0.4	0.0	0.7	0.0575	0.6	− 0.2	1.5	0.1542

*ALCAR* acetyl-l-carnitine, *FVC* forced vital capacity, *ALSFRS-R* amyotrophic lateral sclerosis functional rating scale-revised, *CL* confidence limit

^a^Matched by age (± 5 years), sex, disease duration (± 3 years), site of onset; adjusted by riluzole use, EL Escorial category, progression rate category

### Post-hoc sensitivity analysis: propensity score matching

Results obtained using a propensity score matching were in line with those obtained in the primary analysis (with matching on age, sex, onset and time from diagnosis to baseline) (Table 7). A total of 45 subjects treated with ALCAR 3 g/day and 45 treated with ALACAR 1.5 g/day were matched 1:1 with not treated subjects on propensity score. Covariates used for the calculation of propensity score were well balanced between treated and not treated subjects after matching. When considering subjects treated with ALCAR 3 g/day and their propensity score matched not treated subjects, 46% in both groups died within 24 months after baseline (OR 1.00, 95% CI 0.44–2.29). No statistically significant differences between the two treatment groups were observed in ALSFRS total score, FVC and self-sufficiency (Table 7). At 24 months 28% of subjects treated with ALCAR 1.5 g/day died, as compared to 55% among their propensity score matched not treated subjects (OR 0.33, 95% CI 0.44–2.29). No statistically significant differences between the two treatment groups were observed in ALSFRS total score, FVC and self-sufficiency (Table 7).

**Table 7:** Post-hoc sensitivity analysis: propensity score matching

ALCAR 3 g/day vs. not treated				
	ALCAR	Not treated		
Death	%	%	OR	95% CI
	46	46	1.00	0.44–2.29
ALSFRS-R	Slope	Slope	Delta slope	*p*
	− 1.17	− 1.13	− 0.04	0.8496
FVC	Slope	Slope	Delta slope	*p*
	− 2.43	− 3.04	0.61	0.2274
Non-self-sufficiency	%	%	OR	95% CI
	95	88	2.71	0.50–14.84

ALCAR 1.5 g/day vs. not treated				
	ALCAR	Not treated		
Death	%	%	OR	95% CI
	28	55	0.33	0.14–2.29
ALSFRS-R	Slope	Slope	Delta slope	*p*
	− 1.02	− 1.35	0.33	0.0877
FVC	Slope	Slope	Delta slope	*p*
	− 2.21	− 2.80	0.59	0.1945
Non-self-sufficiency	%	%	OR	95% CI
	95	86	2.84	0.54–15.00

## Discussion

Our findings did not confirm an effect of ALCAR 3 g/day on survival in ALS subjects at 24 months. An effect was observed in those treated with ALCAR 1.5 g/day.

Even though the proportion of patients becoming non-self-sufficient is now a validated endpoint for trials in ALS [9], we decided to use the overall survival as primary endpoint based on the results from a previous clinical trial [11]. Also, in a retrospective observational study it is more feasible to collect survival data rather than repeated measures of ALSFRS-R scores over a long follow-up period.

Regarding the secondary endpoints, we did not obtain significant results in self-sufficiency and FVC with both ALCAR 3 g/day and 1.5 g/day. In subjects treated with ALCAR 1.5 g/day, the difference in ALSFRS-R slope as compared to not treated was 0.4 (95% CI 0.0, 0.7; *p* = 0.0575), while the slopes were virtually the same in treated with 3 g/day and their matched controls.

These data are not in line with the previous findings [11], but this could be probably explained by the retrospective design of the present study, which did not allow a regular collection of ALSFRS-R scores and FVC% at fixed time points.

In the previous pilot clinical trial, an effect on survival was observed using a dosage of 3 g/day, while in our observational study, the effective dose was 1.5 g/day and, unexpectedly, no effect was observed in subjects treated with 3 g/day. In addition, we did not detect an effect on self-sufficiency at 12 months as previously seen in the pilot trial. These differences could be explained by the study design (retrospective observational study vs prospective randomized trial), selection bias (subjects from the real-world clinical practice are less selected than those included in a clinical trial), drug compliance (subjects enrolled in a clinical trial perform several on site evaluation in which compliance is verified by tablets accounting, while in clinical practice this is not done).

As observed in Herzmann [14], the mean of the plasmatic levels of ALCAR were significantly higher in patients with confirmed HIV-1 infection treated with ALCAR at different dosages than in the control group. In the same study, Herzmann observed that plasmatic levels of ALCAR did not differ between different daily dosage regimens. However, intra-mitochondrial levels remain unknown and the pharmacokinetic profile of orally administered ALCAR is complex and likely to be affected by endogenous concentrations. Based on this observation, the differences between the two dosages observed in our study are not expected to be justified by ALCAR plasmatic levels.

Instead, the observed differences could be explained by the incomplete control of the disease heterogeneity in an observational study. Randomization is the unique instrument able to achieve an effective balance of all confounding factors between groups. Differences in the effect of the two dosages could in part be explained by baseline imbalance in the El Escorial category, progression rate and usage of riluzole. We adjusted all analyses by these variables but this may not have been sufficient to completely control their possible confounding effect.

Our hypothesis is that the presence of residual confounding might explain our unexpected results. Residual confounding refers to the presence of an unmeasured or uncontrolled variable that could affect the relationship between treatment (ALCAR) and outcome. Observational studies are prone to residual confounding because they do not involve random assignment of participants to treatment and control group. Therefore, the positive results observed in the group treated with ALCAR 1.5 g/day may be due to some unmeasured or uncontrolled variable. Also, unmeasurable or uncontrolled confounding could have determined the absence of effect in the ALCAR 3 g/day group.

This study has several strengths. First, it included treatment groups that were well-balanced on the most relevant prognostic factors. The matching of subjects helped reducing the impact of potential confounding variables and strengthens the internal validity of the study. Second, each subject was followed for a minimum of 24 months or until death. This allowed a long-term evaluation of the treatment effect. Third, we chose to use overall survival as the primary endpoint, which is a hard outcome, providing a comprehensive assessment of the treatment effect in a retrospective study, although the proportion of patients becoming non-self-sufficient and ALSFRS-R total score, used as secondary outcomes, are validated endpoints for trials in ALS. At last, tertiary centres from several Italian regions were included and subjects included in the study should be representative of the Italian general ALS patients population. This should increase the external validity of the study.

The study has some limitations. This is a retrospective study and no data regarding cognitive impairments nor comorbidities nor risk factors were available; no biological data were available to test differences in biomarkers evolution and genetic mutations; inter-rater agreement on the use of ALSFRS-R was not performed and this could have affected the consistency of the evaluation between centres. As this is an observational study (not randomized) we cannot exclude the presence of residual confounding. To evaluate the impact of a different matching approach, we performed a post-hoc sensitivity analysis using a propensity score matching. Propensity score matching simulate the effects of randomization in observational studies. All measured baseline covariates were well balanced between groups after propensity score matching and the results obtained were in line with those obtained in the primary analysis. This confirms that variables selected for matching (age, sex, time from diagnosis to baseline, onset), in combination with multivariable adjustment for unbalanced covariates in the primary analysis, allowed a good control of confounding. However, this is not sufficient to exclude the possibility of residual confounding due to unmeasured or even unmeasurable baseline covariates having an impact on treatment assignment and outcome. The last limitation is that, even if the sample size was adequate for the evaluation of our primary endpoint, this was not sufficient to perform adequately powered subgroup analyses (by onset, progression rate, age, sex).

This study provided additional information on the potential effect of ALCAR on disease progression and survival and adds evidence to justify the use of ALCAR in ALS subjects. In addition, our results underlined that ALCAR has a good safety profile in people living with ALS in the context of real use outside clinical trials. A difference on survival was detected between ALCAR 1.5 g/day and not treated subjects. As already discussed, ALCAR is prescribed at different dosages in Italy, but no investigation was performed to select the most efficient dosage and hopefully to understand if different dosages are requested based on disease progression. At this point, we suggest to better investigate the effect of different dosages by a randomized, double-blind placebo-controlled, longitudinal prospective trial, with three arms (placebo, ALCAR 3 g/day, ALCAR 1.5 g/day) and long-term follow up (12 months). Such a study will evaluate the effect of ALCAR 1.5 g/day or 3 g/day removing the confounding elements (which affects observational studies) through randomization.

## Data Availability

Anonymized data will be shared by request from any qualified investigator.
