# Correction to: Retrospective observational study on the use of acetyl-l-carnitine in ALS

**DOI:** 10.1007/s00415-023-11960-3

**Published:** 2023-09-08

**Authors:** Serena Sassi, Elisa Bianchi, Luca Diamanti, Danilo Tornabene, Elisabetta Sette, Doriana Medici, Sabrina Matà, Deborah Leccese, Martina Sperti, Ilaria Martinelli, Andrea Ghezzi, Jessica Mandrioli, Valentina Virginia Iuzzolino, Raffaele Dubbioso, Francesca Trojsi, Carla Passaniti, Giulia D’Alvano, Massimiliano Filosto, Alessandro Padovani, Letizia Mazzini, Fabiola De Marchi, Lucia Zinno, Andi Nuredini, Paolo Bongioanni, Cristina Dolciotti, Elena Canali, Giulia Toschi, Antonio Petrucci, Alessia Perna, Vittorio Riso, Maurizio Inghilleri, Laura Libonati, Chiara Cambieri, Elisabetta Pupillo

**Affiliations:** 1 Istituto di Ricerche Farmacologiche Mario Negri IRCCS, Milan, Italy; 2 Neuro-Oncology Unit, IRCCS Mondino Foundation, Pavia, Italy; 3 Department of Brain and Behavioral Sciences, University of Pavia, Pavia, Italy; 4 UO di Neurologia Dipartimento Neuroscienze e Riabilitazione, Azienda Ospedaliera Universitaria di Ferrara, Ferrara, Italy; 5 Presidio Ospedaliero Fidenza AUSL Parma, Fidenza, Italy; 6 Dipartimento Neuromuscoloscheletrico e Degli Organi di Senso, Azienda Ospedaliero-Universitaria di Careggi, Florence, Italy; 7 Department of Neurosciences, Azienda Ospedaliero Universitaria di Modena, Modena, Italy; 8 Clinical and Experimental Medicine PhD Program, University of Modena and Reggio Emilia, Modena, Italy; 9 Department of Biomedical, Metabolic and Neural Sciences, University of Modena and Reggio Emilia, Modena, Italy; 10 Department of Biomedical, Metabolic and Neural Sciences, Centre for Neuroscience and Nanotechnology, University of Modena and Reggio Emilia, Modena, Italy; 11 Department of Neurosciences, Reproductive Sciences and Odontostomatology, University of Naples Federico II, Naples, Italy; 12 Dipartimento di Scienze Mediche e Chirurgiche Avanzate, Università degli Studi della Campania “Luigi Vanvitelli”, P.Zza Miraglia 2, Naples, Italy; 13 Department of Clinical and Experimental Sciences, NeMO-Brescia Clinical Center for Neuromuscular Diseases, University of Brescia, Brescia, Italy; 14 Department of Clinical and Experimental Sciences, Unit of Neurology ASST Spedali Civili Di Brescia, University of Brescia, Brescia, Italy; 15 ALS Center Azienda Ospedaliero Universitaria “Maggiore della Carità” e Università del Piemonte Orientale, Novara, Italy; 16 Department of Medicine and Surgery, University of Parma, Parma, Italy; 17 Dpt. Medical Specialties, Azienda Ospedaliero-Universitaria Pisana, Pisa, Italy; 18 U.O di Neurologia, Presidio Ospedaliero S.Maria Nuova Azienda USL, IRCCS di Reggio Emilia, Florence, Italy; 19 Center for Neuromuscular and Neurological Rare Diseases, San Camillo Forlanini Hospital, Rome, Italy; 20 Dipartimento di Neuroscienze Umane, Università di Roma “Sapienza” UOSD Malattie Neurodegenerative-Centro Malattie Rare Neuromuscolari, Policlinico Universitario Umberto I, Rome, Italy

**Correction to: Journal of Neurology** 10.1007/s00415-023-11844-6

The original version of this article unfortunately contained a mistake. The acknowledgements section was incorrect.

The correct acknowledgement section should read as

Massimiliano Filosto, Francesca Trojsi, Letizia Mazzini. Fabiola De Marchi, Raffaele Dubbioso and Valentina Virginia Iuzzolino are members of the European Reference Network Euro-NMD. All authors are member of the MND Italian Study Group.

The original article has been corrected.

